# Gender, Vertical Height and Horizontal Distance Effects on Single-Leg Landing Kinematics: Implications for Risk of non-contact ACL Injury

**DOI:** 10.2478/hukin-2013-0022

**Published:** 2013-07-05

**Authors:** Nicholas Ali, Gholamreza Rouhi, Gordon Robertson

**Affiliations:** 1 School of Human Kinetics, University of Ottawa.; 2 Department of Mechanical Engineering, University of Ottawa.

**Keywords:** Injury mechanism, kinematics, kinetics, ground reaction force (GRF), knee abduction moment, risk factors

## Abstract

There is a lack of studies investigating gender differences in whole-body kinematics during single-leg landings from increasing vertical heights and horizontal distances. This study determined the main effects and interactions of gender, vertical height, and horizontal distance on whole-body joint kinematics during single-leg landings, and established whether these findings could explain the gender disparity in non-contact anterior cruciate ligament (ACL) injury rate. Recreationally active males (n=6) and females (n=6) performed single-leg landings from a takeoff deck of vertical height of 20, 40, and 60 cm placed at a horizontal distance of 30, 50 and 70 cm from the edge of a force platform, while 3D kinematics and kinetics were simultaneously measured. It was determined that peak vertical ground reaction force (VGRF) and the ankle flexion angle exhibited significant gender differences (p=0.028, partial η ^2^ =0.40 and p=0.035, partial η ^2^ =0.37, respectively). Peak VGRF was significantly correlated to the ankle flexion angle (r= −0.59, p=0.04), hip flexion angle (r= −0.74, p=0.006), and trunk flexion angle (r= −0.59, p=0.045). Peak posterior ground reaction force (PGRF) was significantly correlated to the ankle flexion angle (r= −0.56, p=0.035), while peak knee abduction moment was significantly correlated to the knee flexion angle (r= −0.64, p=0.03). Rearfoot landings may explain the higher ACL injury rate among females. Higher plantar-flexed ankle, hip, and trunk flexion angles were associated with lower peak ground reaction forces, while higher knee flexion angle was associated with lower peak knee abduction moment, and these kinematics implicate reduced risk of non-contact ACL injury.

## Introduction

Most non-contact ACL injuries occur during activities involving single-leg landings ( Boden et al., 2009 ). In a jump landing event, the landing phase is more stressful to the ACL than takeoff ( Chappell et al., 2002 ). Single-leg landings are common tasks performed from varying vertical heights and horizontal distances during sporting events such as volleyball, basketball and soccer ( Dufek and Bates, 1991 ). The literature informs us that single-leg and double-leg landing biomechanics are different ( McNitt-Gray, 1993 ). Single-leg landings result in greater risk of non-contact ACL injuries compared to double-leg landings ( Pappas et al., 2007 ; Yeow et al., 2010 ). There are many single-leg landing studies in the literature ( Self and Paine, 2001 ; Lephart et al., 2002 ; Fagenbaum and Darling, 2003 ; Hargrave et al., 2003 ; Ford et al., 2006 ; Russell et al., 2006 ; Nagano et al., 2007 ; Pappas et al., 2007 ; Schmitz et al., 2007 ; Lawrence III et al., 2008 ; Kiriyama et al., 2009 ; Shimokochi et al., 2009 ; Yeow et al., 2010 ). These studies explicate factors implicated in contributing to the risk of non-contact ACL injuries, as well as, biomechanical gender differences that possibly explain the gender disparity in non-contact ACL injury rate ( Fagenbaum and Darling, 2003 ; Ford et al., 2006 ; Russell et al., 2006 ; Nagano et al., 2007 ; Pappas et al., 2007 ; Schmitz et al., 2007 ; Lawrence III et al., 2008 ; Kiriyama et al., 2009 ; Shimokochi et al., 2009 ; Yeow et al., 2010 ). However, most of these studies investigated single-leg landings from only one vertical height ( Self and Paine, 2001 ; Lephart et al., 2002 ; Hargrave et al., 2003 ; Ford et al., 2006 ; Russell et al., 2006 ; Nagano et al., 2007 ; Pappas et al., 2007 ; Schmitz et al., 2007 ; Lawrence III et al., 2008 ; Kiriyama et al., 2009 ; Shimokochi et al., 2009 ) and to the authors’ best knowledge, none of these studies investigated the effect of horizontal distance on single-leg landing biomechanics. Moreover, many of these studies investigated vertical heights lower than 30 cm, which as argued by Zhang et al. (2000) is too low, and the participant’s landing strategies may be completely different at higher heights. In addition, to the authors’ best knowledge none of these studies investigated the interaction of gender, vertical height, and horizontal distance on single-leg landing kinematics. As well, we observed that some single-leg landing studies only report data on knee kinematics ( Fagenbaum and Darling, 2003 ; Hargrave et al., 2003 ; Russell et al., 2006 ; Nagano et al., 2007 ; Pappas et al., 2007 ), but it may be equally important to simultaneously consider the role of ankle, knee, hip and trunk kinematics as they act in concert to modulate impact forces. Apart from the knee joint, the trunk, hip and ankle joint may also contribute to the overall shock absorption through their respective flexion motions during landing ( Zhang et al., 2000 ).

Gender differences in whole-body joint kinematics and how these kinematics can attenuate GRFs and knee abduction moments (to mitigate the risk of non-contact ACL injury) during single-leg landings from increasing vertical heights and horizontal distances are not yet known. The current study examines the relationships between three non-contact ACL injury risk predictor variables, namely, peak vertical ground reaction force (VGRF), peak posterior ground reaction force (PGRF), and peak knee abduction moment, and various single-leg landing biomechanical variables. These three non-contact ACL injury risk predictor variables were selected for the following reasons. Firstly, an *in vivo* study demonstrated for a male subject hopping and landing on a single leg, the peak in vivo ACL strain occurred at the same instant as peak VGRF, suggesting that peak VGRF may predict the risk of non-contact ACL injury ( Cerulli et al., 2003 ). Other studies have also determined that landing with a high impact force may pose high risk to the ACL ( Chappell et al., 2002 ; Boden et al., 2009 ). Secondly, it was shown that an increase in peak PGRF requires an increase in knee extensor moment for balance, and this knee extensor moment generated by the quadriceps muscle was a major contributor to the higher proximal tibia anterior shear forces that likely increases ACL loads ( Yu and Garrett, 2007 ). Therefore, peak PGRF may also predict the risk of non-contact ACL injury. Finally, the literature has shown that knee abduction moment may also predict the risk of non-contact ACL injury ( Hewett et al., 2005 ). The objective of this study was twofold: first, to examine the main effects and interactions of gender, vertical height, and horizontal distance on whole-body joint kinematics during single-leg landings; and second, to correlate the biomechanical variables significantly impacted by main effects and interactions of gender, vertical height and/or horizontal distance to three non-contact ACL injury risk predictor variables. It was hypothesized that males and females would demonstrate significantly different whole-body joint kinematics, which would explain the higher incidences of non-contact ACL injury among females.

## Material and Methods

### Participants

Six male recreational athletes with a mean age of 24.7 ± 1.9 years, body height of 172 ± 11 cm, and body mass of 69.5 ± 8.6 kg, and six female recreational athletes with a mean age of 23.3 ± 1.86 years, body height of 170 ± 3 cm, and body mass of 66.75 ± 6.2 kg, were recruited from the university population. None of the participants reported any previous history of musculoskeletal, ligamentous or orthopaedic injuries to the lower extremity at the time of participation. A recreational athlete was defined as a participant who takes part in some form of a jump landing sport for 30 minutes a day at least 3 times a week. Prior to data collection, each participant gave informed consent as stipulated by the university’s ethics review board. Participants’ age and anthropometrics were recorded. The dominant leg was established as the leg used by the participant to kick a ball.

### Procedures

**Figure 1:**
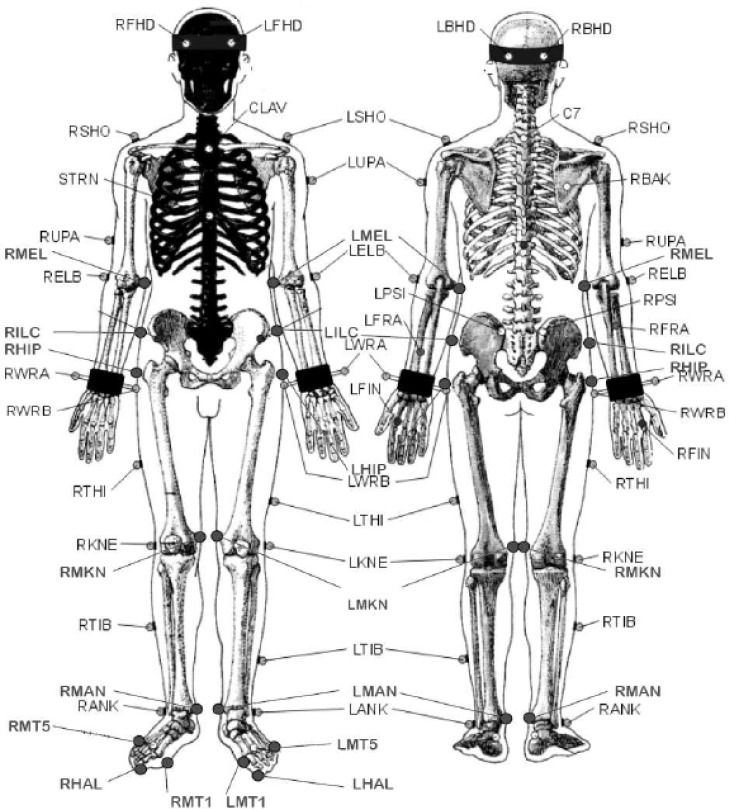
*
Customized marker set used in this study.
* * *Adapted from Oxford Metrics Plug-in-gait marker placement document
*

All participants wore identical shoes (running shoe, model BY004, ASICS America Corporation, Irvine, CA) throughout data collection, so as to mitigate possible variability. Retro-reflective markers were fixed with a double-sided tape using a customized version of Vicon Plug-in Gait marker set ( Figure 1 ). The Vicon Plug-in Gait marker set was customized to include additional markers at the hip and medial aspects of the elbow, knee and ankle as well as additional foot markers. Different marker locations were also used at the proximal ends of the pelvis. A total of 42 retro-reflective markers were used on each participant. A seven-camera motion capture system (Vicon MX, Oxford Metrics, UK) collected marker trajectories at a sampling rate of 250 Hz. A force plate (Kistler type 9281B, Winterthur, Switzerland) measured GRFs at a sampling rate of 1000 Hz. Videographic and force plate data were time synchronized. The VGRF was defined as the reaction to the force the body exerts on the ground in the vertical direction. The PGRF was defined as the horizontal reaction force the body exerts on the ground in the backward direction from landing.

Before data collection, each participant was given enough time to warm-up and practice the single-leg landing task until comfortable. The command of ‘ready’ was given to the participants before the start of each landing task. For each landing task all participants began in a standard take-off position by standing on a takeoff deck with hands placed on the iliac crests, legs shoulder width apart, and the toes of both feet aligned with the edge of the deck. Participants were then instructed to stand on their dominant leg, jump forward, and land as naturally as possible with their dominant foot only centered on the force plate. The participants were asked to keep their hands on their iliac crests when landing to reduce any variability from swinging arms. The participants were instructed to perform single-leg landings from takeoff decks of three different vertical heights (20, 40, and 60 cm) that were placed at three horizontal distances (30, 50 and 70 cm) from the edge of a force plate. The combination of vertical height and horizontal distance was defined as a landing configuration. The nine different landing configurations tested were h20d30, h20d50, h20d70, h40d30, h40d50, h40d70, h60d30, h60d50, and h60d70, where h represents the vertical height and d represents the horizontal distance. The number after h and d refers to the vertical height and horizontal distance, respectively, in centimeters. Each participant performed two trials at each landing configuration. The sequence of landing configurations were randomized to reduce learning effects.

### Data reduction and analysis

One trial was selected from the better of two trials for model building, data analysis, and reporting. In general, each participant performed two trials at each landing configuration. The better trial was determined as the one in which the participant did not remove their hands from the iliac crests during landing, did not allow their non-dominant leg to impact the force plate during landing, or did not lose a marker during impact with the ground. At the time of peak VGRF, joint kinematics and kinetics were determined for the dominant leg. All marker trajectories and analog data were imported into Visual3D (C-Motion Inc. Rockville, MD) biomechanical software. In Visual3D, ankle dorsiflexion was defined as positive, ankle plantar flexion as negative, knee flexion as negative, hip flexion as positive, and trunk flexion as positive. The ankle flexion angle was defined as the angle between the leg segment and foot segment. The difference between the ankle flexion angle when the participant stood on the takeoff platform and when peak VGRF occurred during landing was determined as the ankle flexion angle. The knee flexion angle was defined as the angle between the thigh and leg segment, while the hip flexion angle was defined as the angle between the thigh and pelvis segment. Trunk flexion angle was calculated as the angle between the trunk segment and a vertical line in the laboratory coordinate system. Kinematic data were low-pass filtered using a second-order bidirectional Butterworth filter at 6Hz and analog data were filtered at 25Hz. Kinetic data were calculated using a Newtonian inverse dynamics analysis by Visual3D software. The ground reaction forces were normalized to body weight and knee abduction moments normalized by the product of body mass and body height.

### Statistical analysis

Multiple repeated-measures three-way MANOVAs were first conducted to test the main effects and interactions of gender (males and females), vertical height (20, 40 and 60 cm) and horizontal distance (30, 50 and 70 cm) on various single-leg landing biomechanical dependent variables; namely, peak VGRF, peak PGRF, peak knee abduction moment, as well as, ankle, knee, hip and trunk flexion angle. Descriptive statistics for these biomechanical variables are presented. Follow-up tests entailed Pearson Product Moment Correlations (PPMCs) determined for variables significantly impacted by the main effects and interactions of vertical height, horizontal distance and gender. PPMCs were measured to determine the associations between the three non-contact ACL injury risk predictor variables and the biomechanical variables. The α level was set at 0.05 for statistical analyses conducted in SPSS (SPSS for Windows, Release 11.5.0).

## Results

Figures 2a , 2b and 2c show the time histories of VGRFs, PGRFs, and knee abduction moments, respectively, during single-leg landings for a participant at the nine landing configurations tested.

The key findings from the separate ANOVAs conducted are shown in Table 1 . From Table 1 , a significant main effect of gender with peak VGRF (F(1,10)=6.56, p=0.028, partial η ^2^ =0.40, observed power=0.64) and with the ankle plantar/dorsiflexion angle (F(1,10)=5.92, p=0.035, partial η ^2^ =0.37, observed power=0.60) was determined. Females had significantly lower peak VGRF and ankle plantar flexion angles compared to males ( Table 2 ). Among the three ACL injury risk predictor variables, follow-up tests revealed peak VGRF was significantly and negatively correlated to the ankle plantar/dorsiflexion angle among males (r=–0.80, p=0.048), while no significant correlation was observed for females. From Table 1 , we also observed that there was a significant height×distance interaction with peak VGRF (F(4, 40)=4.67, p=0.003; partial η ^2^ =0.32, observed power=0.92), hip flexion angle (F(4, 40)=3.96, p=0.008, η ^2^ =0.28, observed power=0.87) and trunk flexion angle (F(4, 40)=3.90, p=0.022, η ^2^ =0.28, observed power=0.86). There was a significant height×gender interaction with trunk flexion angle (F(2, 20)=7.58, p=0.020, η ^2^ =0.43, observed power=0.91) ( Table 1 ). Results revealed no significant distance×gender or height×distance×gender interactions for any of the biomechanical variables tested ( Table 1 ). Results showed many significant main effects of both vertical height and horizontal distance with the biomechanical variables tested ( Table 1 ). With the exception of peak VGRF and ankle plantar/dorsiflexion angle, given there was no significant effect of gender on any of the other dependent variables tested, the male and female data were pooled for subsequent follow-up statistical testing using PPMCs. Descriptive statistics of the biomechanical variables after pooling male and female data are presented in Table 3 , while PPMCs among the three non-contact ACL injury risk predictor variables and the biomechanical variables tested are presented in Table 4 . From Table 4 , it can be gleaned that peak VGRF was significantly and negatively correlated to the ankle plantar/dorsiflexion angle (r= −0.59, p=0.04), hip flexion angle (r= −0.74, p=0.006), and trunk flexion angle (r= −0.59, p=0.045). We also observed that peak PGRF was significantly and negatively correlated to the ankle plantar/dorsiflexion angle (r= −0.56, p=0.04). Finally, there was a significant and negative correlation between peak knee abduction moment and the knee flexion angle (r= −0.64, p=0.03).

## Discussion

The trends determined in GRFs ( Figure 2a and Figure 2b ) for the single-leg landing tasks performed in the current study did not reveal the characteristic distinct bimodal GRF curve reported for double-leg landings in the literature ( Dufek and Bates, 1990 ; Zhang et al., 2000 ). The single-leg landing tasks in the current study resulted in a smooth and rapid increase in GRFs with a single peak, which is consistent with the findings of Hargrave et al. (2003) . As a result, the biomechanical comparison of double-leg and single-leg landing studies in the literature may be limited due to the differences exhibited by these two tasks.

Safe landing technique usually involves movements that act to dissipate high impact forces. Given that the single-leg landing tasks in the current study were sagittal plane dominant, possibly explains the lack of kinematic gender differences, and perhaps these differences become more pronounced during out-of-plane movements. Out-of-plane motion such as side-step cutting or single-leg landing to the medial or lateral aspect of the knee may entail both sagittal and non-sagittal plane loadings and perhaps pose greater risk to ACL injury. Nonetheless, we observed females landed with significantly lower peak VGRF compared to males, a finding that is not supported by two studies ( Pappas et al., 2007 ; Schmitz et al., 2007 ). In the current study, females may have experienced lower VGRF because of their lower body mass compared to males, and perhaps musculoskeletal differences such as higher quadriceps-to-hamstring ratio, the latter factor not considered in this study. The reason for lower peak VGRF in females during single-leg landings has yet to be elucidated. Ankle plantar/dorsiflexion angle was the only kinematic variable that exhibited significant gender differences with females landing dorsiflexed while males predominantly plantar flexed ( Table 2 ). This finding partly supports our hypothesis and may also partly explain why females are at greater risk of non-contact ACL injury given the literature has shown that athletes who injured their ACL had significantly less plantar flexed ankle angle ( Self and Paine, 2001 ; Madigan and Pidcoe, 2003 ; Boden et al., 2009 ; Shimokochi et al., 2009 ). It has also been shown that kinematic differences at the ankle may contribute to gender differences in the ACL injury rate ( Griffin et al., 2000 ). The results of the current study are also consistent with the literature that reported no gender differences during single-leg landings in hip flexion angles ( Lephart et al., 2002 ; Pappas et al., 2007 ), and knee flexion angles ( Nagano et al., 2007 ; Kiriyama et al., 2009 ). As well, the current study found a significant and negative correlation between peak VGRF and ankle flexion angle for males, a finding that is supported by the literature ( Self and Paine, 2001 ; Madigan and Pidcoe, 2003 ; Boden et al., 2009 ; Shimokochi et al., 2009 ). Perhaps higher ankle plantar flexion angles permit more time to distribute the impact forces and better enable the musculature to dissipate these forces as demonstrated by McNitt-Gray (1993) .

Table 4 showed that higher ankle plantar flexion, hip, and trunk flexion angles are associated with lower peak VGRF. Our finding that higher hip flexion angle was associated with lower peak VGRF is corroborated by the literature ( Ball, 1999 ; Self and Paine, 2001 ). Furthermore, our findings are consistent with that of Ball (1999) who showed lower hip flexion angles pose greater risk of ACL injury; given that the quadriceps’ high compensatory knee torques acting in combination with GRFs may excessively accelerate the tibia anteriorly beneath the femur. To the authors’ best knowledge, no single-leg landing studies have investigated trunk flexion angles; therefore, direct comparison of our results with the literature was not possible. However, the findings from the current study are consistent with a double-leg landing study ( McNitt-Gray, 1993 ) that showed that higher trunk flexion angle was associated with lower peak VGRF. This finding is also in general agreement with a study by Hewett’s research group that recommended participants land with their chest over their knees to reduce the likelihood of non-contact ACL injury ( Hewett et al., 2009 ). From Table 4 we also observed that lower peak knee abduction moment was associated with higher knee flexion angles, a finding that is supported by the literature ( Hewett et al., 2005 ). Higher plantar flexed ankle, hip, and trunk flexion angles, as well as, higher knee flexion angles during single-leg landings reduce peak GRFs and the peak knee abduction moment, respectively. These kinematics implicate reduced risk of non-contact ACL injury that is corroborated by the literature ( Alentorn-Geli et al., 2009 ).

Even though the current study used a small sample size (n=12), many significant main effects and interactions with high statistical power, as well as, medium to high Pearson correlation coefficients were observed, suggesting the sample size used was adequate. This may be attributed to our experimental design that used large differences in task demands, our application of repeated-measures MANOVAs, and employment of homogenous data (body height and body mass matched males and females). While we cannot conclude (given small sample size) that the general male or female population would exhibit whole-body kinematics as reported in the current study, we were able to show that the relationships found have a strong support in terms of partial η ^2^ , r ^2^ and p values for the participants tested. In addition, even though reasonable correlations were obtained, this study was performed in a controlled laboratory environment where participants could plan for these tasks and as such, may not be representative of maneuvers experienced during sports. As well, the relatively small sample size used in the current study may have hindered our ability to detect small but significant differences among the variables tested. In addition, this study did not address the ability of the musculoskeletal system to absorb energy upon impact, even though a study ( Lees, 1981 ) showed that muscular activity during landing can modify GRFs. Furthermore, this study did not investigate the anatomical and hormonal factors implicated to increase the risk of non-contact ACL injuries. Even though single-leg landings performed in the current study were sagittal plane dominant, out-of-plane movements commonly involved in sports and not captured in this study may be important contributors to the risk of non-contact ACL injury. Therefore, future studies should investigate the biomechanical demands of frontal and traverse plane loading during out-of-plane landings on a single leg. Additionally, this study may be limited given there is equally a limited number of studies supporting the use of peak VGRF, peak PGRF, and peak knee abduction moment as ACL injury risk predictor variables. Since we did not measure ACL loads during single-leg landings, the results and discussions stemming from the current study are based on what is known about the relationship between ACL loads and GRFs, as well as, ACL loads and knee abduction moment.

## Conclusions

Within the findings and limitations of the current study, peak VGRF and ankle flexion angle were the only variables that demonstrated significant gender differences during single-leg landings from increasing vertical heights and horizontal distances. Females had significantly lower peak VGRF and the ankle plantar flexion angle during single-leg landings, in which the latter may have hindered their ability to attenuate GRFs, and may partly explain why this gender is at greater risk of non-contact ACL injury. Higher plantar flexed ankle, hip, and trunk flexion angles were associated with lower peak GRFs while higher knee flexion angle was associated with lower peak knee abduction moment. Higher values of these kinematic variables during single-leg landings suggest modulation of GRFs and knee abduction moments, which implicates reduced risk of non-contact ACL injury.

## Figures and Tables

**Figure 2a:**
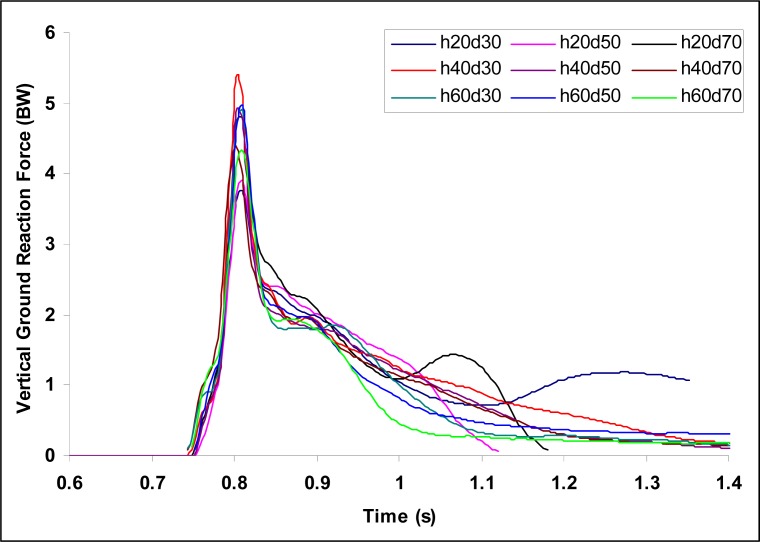
*
Time histories of VGRFs during single-leg landings from the nine landing configurations for a participant.
*

**Figure 2b:**
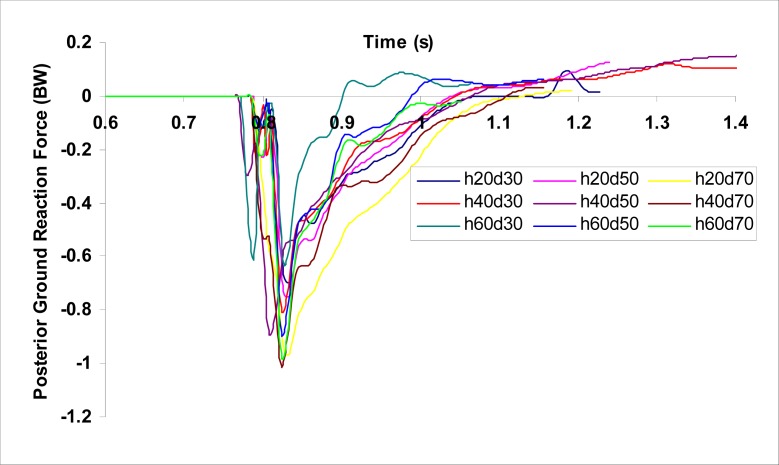
*
Time histories of PGRFs during single-leg landings from the nine landing configurations for a participant.
*

**Figure 2c:**
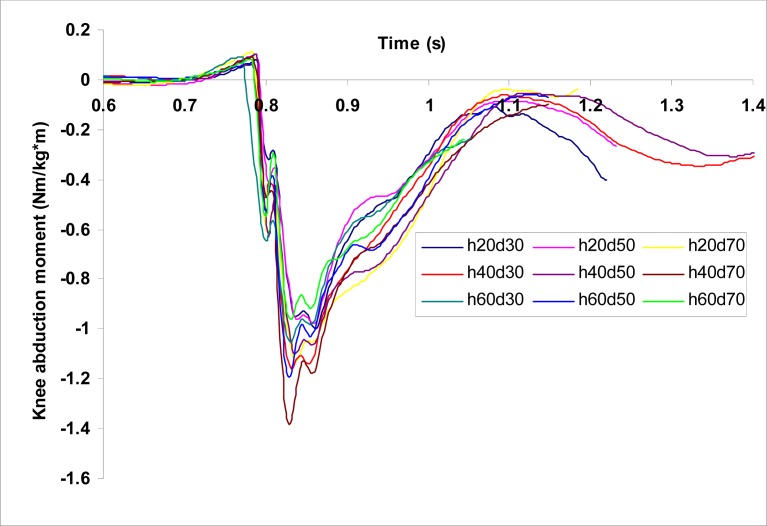
*
Time histories of knee abduction moments during single-leg landings from the nine landing configurations for a participant.
*

**Table 1:** *
ANOVA summary showing interactions and main effects observed.
*

	Dependent Variables						
	Peak VGRF	Peak PGRF	Ankle plantar/dorsi flexion angle	Knee flexion angle	Peak Knee abduction moment	Hip flexion angle	Trunk flexion angle
Height	0.000	0.000		0.000			0.040
Distance		0.000	0.000		0.007	0.000	
Height x Gender							0.020
Distance x Gender							
Height x Distance	0.003					0.008	0.022
Height x Distance x Gender							
Gender	0.028		0.035				

**Table 2:** *
Descriptive statistics (mean ± SD) of biomechanical variables significantly different between genders.
*

	** Males (n=6) **								

Dependent Variables	h20d30	h20d50	h20d70	h40d30	h40d50	h40d70	h60d30	h60d50	h60d70

Peak VGRF (BW)	3.60±0.39	3.78±0.40	3.91±0.70	5.22±0.34	4.88±0.86	5.29±0.92	5.94±0.73	6.07±0.78	5.56±1.09
Ankle plantar/dorsi flexion angle (deg)	−1.81±5.02	0.93±5.16	2.35±6.86	−4.47±3.41	−2.17±2.16	1.22±5.64	−4.29±4.74	−0.11±3.53	−1.26±5.75

	** Females (n=6) **								

Dependent Variables	h20d30	h20d50	h20d70	h40d30	h40d50	h40d70	h60d30	h60d50	h60d70

Peak VGRF (BW)	3.00±0.29	3.28±0.38	3.46±0.44	4.51±0.24	4.38±0.34	4.63±0.69	5.62±0.75	5.06±0.55	4.89±0.50
Ankle plantar/dorsi flexion angle (deg)	0.94±5.41	2.81±1.88	7.75±6.69	0.59±3.77	5.56±6.94	6.30±9.64	0.73±4.13	3.55±3.73	7.23±3.56

**Table 3:** *
Descriptive statistics (mean ±SD) of the biomechanical dependent variables tested.
*

	** (n=12) **								

** Dependent Variables **	** h20d30 **	** h20d50 **	** h20d70 **	** h40d30 **	** h40d50 **	** h40d70 **	** h60d30 **	** h60d50 **	** h60d70 **
Peak VGRF (BW)	3.30±0.45	3.52±0.46	3.67±0.60	4.87±0.47	4.63±0.68	4.96±0.84	5.78±0.72	5.56±0.83	5.23±0.88
Peak PGRF (BW)	−0.19±0.15	−0.28±0.21	−0.43±0.23	−0.09±0.20	−0.06±0.20	−0.24±0.18	−0.33±0.28	−0.05±0.19	−0.04±0.30
Peak knee abd. moment (N·m/kg·m)	−0.13±0.18	−0.09±0.37	−0.15±0.21	−0.11±0.19	−0.16±0.20	−0.02±0.28	−0.19±0.30	−0.16±0.17	−0.04±0.15
Ankle plantar/dorsiflexion angle (deg)	−0.44±5.10	1.87±3.81	5.05±7.00	−1.94±4.28	1.69±6.30	3.76±7.89	−1.78±4.90	1.72±3.89	2.98±6.26
Knee flexion angle (deg)	−27.86±9.71	−27.04±9.10	−25.91±8.02	−30.44±9.70	−29.55±8.70	−29.17±9.57	31.39±11.09	−31.67±8.10	−30.95±8.85
Hip flexion angle (deg)	21.45±11.30	20.61±10.40	22.62±10.37	20.38±11.20	22.20±10.79	24.26±10.50	17.84±14.10	25.16±10.21	24.88±9.27
Trunk flexion angle (deg)	17.63±6.80	18.1±8.42	21.53±8.45	17.94±9.10	19.61±11.45	17.87±11.00	21.88±9.30	20.52±11.40	20.18±9.60

**Table 4:** *
Bivariate Pearson’s r correlations between the three ACL injury risk predictor variables and biomechanical variables significantly impacted by main effects and interactions of height and distance.
*

** (n=12) **							
** Pearson's Correlation **	** Peak VGRF **	** Peak PGRF **	** Peak knee abduction moment **	** Ankle plantar/dorsi flexion angle **	** Knee flexion angle **	** Hip flexion angle **	** Trunk flexion angle **
Peak VGRF (BW)	1.00	0.18	−0.05	−0.59 *	0.35	−0.74 **	−0.59 *
Peak PGRF (BW)		1.00	−0.09	−0.56 *	0.01	−0.27	0.38
Peak knee abd. moment (N.m/kg.m)			1.00	0.09	−0.64 *	−0.08	0.18
Ankle plantar/dorsiflexion angle (deg)				1.00	0.12	0.58 *	0.54 *
Knee flexion angle (deg)					1.00	0.41	0.13
Hip flexion angle (deg)						1.00	0.45
Trunk flexion angle (deg)							1.00

Note:

* p < 0.05;

** p < 0.01
